# Whooping Cough Cases Increase in Central Italy after COVID-19 Pandemic

**DOI:** 10.3390/antibiotics13050464

**Published:** 2024-05-19

**Authors:** Giulia Linardos, Luana Coltella, Stefania Ranno, Velia Chiara Di Maio, Luna Colagrossi, Elisabetta Pandolfi, Maria Beatrice Chiarini Testa, Leonardo Genuini, Francesca Stoppa, Matteo Di Nardo, Annalisa Grandin, Renato Cutrera, Corrado Cecchetti, Alberto Villani, Massimiliano Raponi, Paola Bernaschi, Cristina Russo, Carlo Federico Perno, Rossana Scutari

**Affiliations:** 1 Unit of Microbiology and Diagnostic Immunology, Bambino Gesù Children’s Hospital, IRCSS, 00165 Rome, Italy; giulia.linardos@opbg.net (G.L.); luana.coltella@opbg.net (L.C.);; 2 Preventive and Predictive Medicine Research Unit, Bambino Gesù Children’s Hospital, IRCSS, 00165 Rome, Italy; 3 Pediatric Pulmonology and Cystic Fibrosis Unit, Bambino Gesù Children’s Hospital, IRCSS, 00165 Rome, Italy; 4 Pediatric Intensive Care Unit, Bambino Gesù Children’s Hospital, IRCCS, 00165 Rome, Italy; 5 General Pediatric and Infectious Disease Unit, Bambino Gesù Children’s Hospital, IRCSS, 00165 Rome, Italy; 6 General Pediatric and Infectious Disease Unit, Pediatric Emergency Medicine, Bambino Gesù Children’s Hospital, IRCCS, 00165 Rome, Italy; 7 Medical Direction, Bambino Gesù Children’s Hospital, IRCCS, 00165 Rome, Italy; 8 Multimodal Laboratory Research Unit, Bambino Gesù Children’s Hospital, IRCCS, 00165 Rome, Italy; rossana.scutari@opbg.net

**Keywords:** *Bordetella pertussis*, whooping cough, infants, epidemiology, vaccine, pregnancy, surveillance

## Abstract

Pertussis continues to be a highly contagious respiratory infection, especially in children, with cyclical peaks of disease spread every three to five years. Here, we report relevant cases of *B. pertussis* infection between August 2023 and January 2024, and compare them with *B. pertussis* prevalence in pediatric patients admitted to the Reference Italian Pediatric Hospital, located in Rome, from January 2015 to July 2023. A total of 5464 tests for *B. pertussis* were performed during the study period, and 6.9% were positive. At the time of the COVID-19 pandemic, there was a sharp decrease in the presence of *B. pertussis*, which reappeared only in August 2023, recording five new cases. All five children presented with paroxysmal cough 5 to 10 days before admission. Four patients had other mild respiratory symptoms and moderate *B. pertussis* DNA levels (Ct mean: 26). Only one child, with very high *B. pertussis* DNA levels (Ct: 9), presented with severe respiratory failure. The patients with mild/moderate infection achieved clinical recovery while the patient with the severe manifestation died of cardiac arrest. These observations highlight the reemergence of pertussis even in vaccinated countries and its association with morbidity and mortality especially in young children. This emphasizes the importance of rapid diagnosis to immediately implement appropriate treatment and monitoring of immune status.

## 1. Introduction

Pertussis (whooping cough), caused by *Bordetella pertussis (B. pertussis)*, is a highly contagious and severe acute respiratory disease in individuals of all ages, most dangerous for young children, especially infants [1]. The classic symptoms are catarrhal and paroxysmal coughs, but in neonates and infants they may also frequently be associated with leukocytosis, pulmonary hypertension, and hypoxemia [2]. An increase in white blood cells typically occurs in cases of severe pertussis, which is defined by pneumonia with refractory hypoxemia and cardiogenic shock [3,4]. Although pertussis is a common infection worldwide, reports of invasive pertussis in the bloodstream to date are rare and have involved immunocompromised patients [5]. This infection can be under-diagnosed, especially during the winter period because it is possible to confuse whooping cough symptoms with those of other common respiratory infections [6]. In fact, evidence confirms the presence of coinfections between *B. pertussis* and other pathogens, particularly viruses, although the impact of these on clinical outcome remains unclear. Some studies have observed a positive association between *B. pertussis* with viral co-infection and the development of pneumonia, but not with severe disease [7,8].

Prevention of pertussis is based on the administration of an acellular pertussis vaccine composed of purified components of the organism *Bordetella pertussis* and detoxified pertussis toxin [9]. In Italy, since 2002, the diphtheria, pertussis, and tetanus (dTp) vaccine has been mandatory in the first year of life; in addition to this, the dTp booster has also been recommended in the third trimester of pregnancy. Nevertheless, pertussis remains an endemic disease worldwide with cyclical peaks of disease spread every three to five years. According to the European Centre for Disease Prevention and Control (ECDC), in 2022 there were more than 2623 cases of pertussis in Europe [4], with one death. Of them, 62 cases were recorded in Italy.

In this study, we report *B. pertussis* prevalence in pediatric patients admitted to the Reference Italian Pediatric Hospital, located in Rome (Italy), from January 2015 to January 2024, and focused on five new relevant cases observed between August 2023 and January 2024.

## 2. Results

### 2.1. Epidemiology

During the 9-year observation period, 5464 tests for Bordetella pertussis were performed and 376 (6.9%) positives were observed. The distribution of positive cases involved mostly younger children; indeed, 68.7% of positive cases affected children <1-year-old.

**Figure 1:**
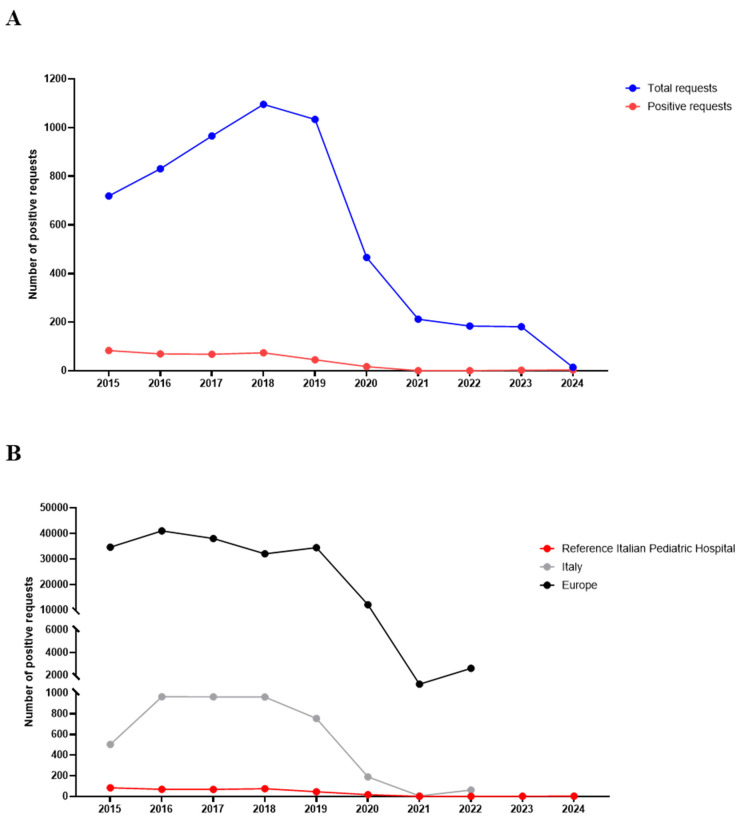
Annual positive cases of *B. pertussis* from 1 January 2015 to 31 January 2024. (**A**) Annual positive cases of *B. pertussis* in patients followed at our Pediatric Hospital. (**B**) Comparison of annual positive cases of *B. pertussis* in patients followed at our Pediatric Hospital, Italy, and Europe.

The total number of pertussis requests and the annual cases of pertussis in patients followed at our hospital from 2015 to 2024 and the comparison of Italian and European data are shown in Figure 1.

Overall, the annual positive cases of pertussis showed little variation over the years observed before the Coronavirus Disease-19 (COVID-19) pandemic (2015–2019). During this period, a sharp decrease in pertussis cases was shown. Notably, the last case of pertussis was reported at the hospital in June 2020, and reappeared again at the end of August 2023. Since its re-emergence, a total of five patients with *B. pertussis* infection have been identified; therefore, we focused on these new cases.

### 2.2. General Patient Characteristics

All pediatric patients under evaluation were Italian with an age of 0.07, 0.08, 0.11, 14 and 16 years. In particular, three of them were neonates and two adolescents (Table 1). All patients were recruited from the emergency department and tested for *B. pertussis* given the presence of recurrent cough.

### 2.3. Microbiology Characteristics

*B. pertussis* DNA was detected in all in respiratory samples by polymerase chain reaction assay, and bacterial growth was observed in three of them on culture examination. Of those patients with B.pertussis detected in culture, colonies were confirmed using MALDI-TOF MS and in all these cases a quantitative culture bacterial showed a concentration of 10^6^ Colony-Forming Unit (CFU). Susceptibility tests showed that all three *B. pertussis* strains isolated were sensitive to the antibiotics tested (azithromycin, erythromycin, clarithromycin, and clindamycin).

At the same time, microbiological investigations based on multiplex PCR, FilmArray, and standard culture tests showed in four out of five patients the presence of at least one other potential pathogen (Table 1). In particular, viral coinfection associated with Human Rhinovirus was shown in one case; Human Rhinovirus/Enterovirus plus Respiratory Syncytial virus was identified in one other patient. A bacterial coinfection characterized by the presence of *Haemophilus influenzae* plus *Staphylococcus aureus* plus *Enterobacter cloacae* was observed in another patient, and a mixed virus/bacteria coinfection was observed in the last patient with Rhinovirus/Enterovirus plus *Staphylococcus aureus* in respiratory samples and *Staphylococcus epidermidis* in blood cultures (Table 1, Supplementary File S1).

### 2.4. Clinical Findings and Outcome

The most common reason for presentation and admission was respiratory symptoms. Fever was reported in only one patient. A typical *B. pertussis* paroxysmal or spasmodic cough that usually ends in a prolonged high-pitched crowing inspiration was present in all children from 5–10 days before admission. Paroxysmal cough was not the only respiratory symptom; indeed, four out five patients (two adolescents and two neonates) were characterized by mild respiratory manifestation. In addition, B.pertussis DNA levels were not too high in these four patients (threshold cycles [CT]: 22, 24, 26, and 30). Three of them, although with normal routine blood tests, C-reactive protein indexes, and pro-calcitonin levels, also required hospitalization (Table 1, Supplementary File S1).

Due to their bacterial infection, all patients received antibiotic treatment based, in most cases, on Azithromycin and Clarithromycin. Depending on the clinical condition, corticosteroid and/or oxygen therapy was added (Table 1, Supplementary File S1).

All four cases were successfully treated and achieved clinical recovery. One of these did not require hospitalization. The other patients were hospitalized for a variable period of 7–26 days; *B. pertussis* DNA was still present at discharge but the culture was negative or not performed.

The fifth patient was characterized by severe manifestation and presented for admission with a very critical clinical condition, including severe respiratory failure (Table 1, Supplementary File S1).

This patient showed a high level of *B. pertussis* DNA (9 CT) and a complex clinical picture characterized by an increase in the white blood cell count equal to 30,000 cells/μL and a decreased lymphocyte count (24.5%) and platelet count (67,000/μL). Furthermore, renal failure was observed with creatinine 1.06 mg/dL, severe hyperkalemia (K 8.1 mEq/L), and hypocalcemia with hyperphosphatemia and elevated uric acid.

Invasive mechanical ventilation with maximal ventilatory parameters, inhaled nitric oxide, and intravenous pulmonary vasodilators were administered for intensive care support, and hemodynamics were supported by adrenaline and noradrenaline. The worsening clinical condition required Extra-Corporeal Membrane Oxygenation (ECMO), and ultrasonography revealed severe biventricular dysfunction, an indirect sign of severe pulmonary hypertension. Despite resuscitation measures, the patient died of cardiac arrest.

Clinical characteristics of patients can be found in Table 1 and in Supplementary File S1.

## 3. Discussion

Whooping cough is an endemic disease worldwide, with widespread peaks of disease every three to five years. In recent years, globally, reported cases of pertussis have changed, mostly associated with the COVID-19 pandemic years. In fact, a decrease in the number of cases was observed during that period, from an average of 434,179 cases in 1980–2019 to 49,587 cases in 2020–2021 [10]. The observed decrease could be due to the lower bacterial circulation caused by the use of personal protective equipment. In contrast, an increase in cases was observed in the last years, reaching 62,646 cases worldwide in 2022 [10]. The increase in the post-pandemic era was also confirmed by data from EU/EEA member states [11]. In line with these observations, the positivity rate also changed over time in our hospital, despite a decrease in specific requests from clinicians to search for *B. pertussis*. In fact, the diagnosis of *B. pertussis* increased by 2.6% (5/195) in the year 2023 (including January 2024) compared with 0% (0/396) observed during the period 2021–2022. This notwithstanding, the observed positivity rate did not match that observed in the 2015–2019 period (7.6%, 339/4467). Notably, the last case of pertussis was reported to the hospital in June 2020, only to reappear at the end of August 2023. All these data point to the resurgence of *B. pertussis* circulation worldwide even in upper-middle-income countries. This could be due to the absence of lockdown and suboptimal adoption of vaccination in newborns during the COVID-19 pandemic. In line with this, according to WHO and UNICEF estimates, globally, ≥1 dose coverage of DTPcv1 increased from 86% in 2021 to 89% in 2022, but remained below the 90% coverage achieved in 2019 [12]. Similarly, the estimated coverage of DTPcv3 increased from 81% in 2021 to 84% in 2022, but remained below the 2019 level (86%) [12]. In the European region, in both 2021 and 2022, coverage of DTPcv1 and DTPcv3 remained ≥97% and ≥94%, respectively [12]. In Italy, from 2000 to 2022, the trend in pertussis vaccination coverage at 24 months of age remained rather unchanged, and never below 93% [13].

In addition, the variation in the positivity rate could be related to the underdiagnosis of *B. pertussis*, either because of the unsolicited specific test for the bacterium or, especially in the winter period, because of the possibility of confusing the symptoms with those of other common respiratory infections. *B. pertussis* infection affects individuals of all ages, producing a spectrum of different clinical manifestations, representing a global public health problem. The severity of the disease in infants is influenced by several factors, including maternal vaccination during pregnancy and the age of the child. In infants, the severity of disease is characterized by the development of pneumonia and/or respiratory insufficiency, which may require therapeutic strategies such as traditional ventilation, high-frequency oscillatory ventilation, plasmapheresis, and ECMO [14]. The worst prognosis is associated with hyperleukocytosis with lymphocyte predominance, so the use of ECMO turns out to be essential by aiming to reduce the number of leukocytes [15,16]. It has been hypothesized that these deaths are due to aggregates of leukocytes in the small pulmonary vessels, resulting in untreatable pulmonary hypertension [17]. Several retrospective studies have established hyperleukocytosis as an indicator of increased mortality in young infants and an independent predictor of mortality in all patients [4,15]. Data from the ECDC_surveillance Atlas of infectious diseases show that the last death case in Italy was recorded in 2014, compared to the European Union which has reported at least one death case per year [13]. Among our reported cases, we describe a fatal case of whooping cough with pulmonary hypertension and respiratory failure that occurred in 2024. The clinical picture observed in our patient is comparable with what was discussed by Halasa et al. regarding four fatal cases of whooping cough in neonates <9 weeks of age who developed pneumonia [18]. Differently, in our case a co-infection with Rhinovirus/Enterovirus and *Staphylococcus epidermidis* was also observed. On one side, this coinfection might have played a role in the clinical complication; nevertheless, several studies have shown so far only positive associations between *B. pertussis* with co-infection and pneumonia, but not with severe disease and outcome [7,8]. Patients with severe clinical situation broad-spectrum antibiotic therapy (prior to any specific diagnosis) should consider drugs that can also control *Bordetella*. In addition to this critical case, we have found other cases of pertussis in the past five months, thus indicating the resurgence of the circulation of this pathogen. In view of the resurgence of *B. pertussis* circulation, probably due to both its classical pattern (3–5 year peaks) and the reduction in preventive measures implemented during the COVID-19 pandemic, it would be appropriate, especially in the case of frail patients (infants and the elderly), to improve prevention measurement and diagnosis for this pathogen. Especially in unvaccinated or partially immunized infants, the source of infection may be contact with a family member, underlining the importance of disease surveillance and revaccination to protect the weaker section of the population [19,20]. Probably the most effective measure to reduce the circulation of pertussis in infants is vaccination of pregnant women in the third month of pregnancy. Vaccination during pregnancy, by stimulating the development of maternal anti-pertussis antibodies, which pass through the placenta, protects infants from pertussis until they are old enough to be vaccinated on their own [21]. In addition, because the symptoms of pertussis overlap with those of acute respiratory viral infections, consideration should be given to implementing the requirement of a specific laboratory test for *B. pertussis* so that the infection is not diagnosed or diagnosed late. This would allow appropriate antibiotic therapy to be implemented early on.

## 4. Materials and Methods

### 4.1. Study Design and Setting

This retrospective study includes patients screened for *Bordetella pertussis* from January 2015 to January 2024 at Italy’s largest reference Pediatric Hospital (about 650 beds), located in Rome.

Nasopharyngeal swabs and nasopharyngeal aspirates were collected from each patient and analyzed at the Microbiology Unit according to standard laboratory operating procedures.

### 4.2. B. pertussis Microbiology

The identification of the *B. pertussis* was performed by specific real-time PCR (Bordetella R-gene^®^, Biomerieux, Marcy-l’Étoile, France) and by a syndromic approach (BioFire^®^ Respiratory 2.1 plus [RP2.1plus] Panel, Biomerieux, Marcy-l’Étoile, France).

Concurrently, *B. pertussis* culture was attempted for all respiratory samples. Specimens were plated on specialized Bordetella-selective agar (Biolife, Milan, Italy) and Bordet Gengou (BD) and plates were incubated under aerobic conditions at 37 °C. Suspected *B. pertussis* colonies were confirmed using MALDI-TOF MS (Bruker, Billerica, MA, USA).

### 4.3. B. pertussis Antimicrobial Susceptibility Test

*B. pertussis* antibiotic susceptibility was analyzed by an E-test. So far, there are no cut-offs for either of the methods recommended by EUCAST, and all determinations for sensitivity or resistance are based on notifications from clinical studies. Antibiotic susceptibility was analyzed against macrolide (azithromycin, erythromycin, and clarithromycin) and lincosamide (clindamycin).

### 4.4. Identification of Other Microorganisms

Several other pathogens were identified by molecular and culture assays. An Allplex Respiratory Panel (Seegene, Seoul, Republic of Korea) and a BioFire FilmArray Respiratory Panel (bioMérieux Clinical Diagnostics, Salt Lake City, UT, USA 2.1) were used to identify primarily the presence of respiratory viruses. Standard growth media for culturing respiratory microorganisms were used for bacteria.

### 4.5. Clinical Information of Post COVID-19 Pandemic B. pertussis Cases

For five pediatric patients diagnosed with pertussis admitted to our hospital from August 2023 to January 2024, age, sex, comorbidities, clinical conditions (body temperature, laboratory tests, and vital parameters), and therapy on the day of admission were collected.

Demographic and clinical information for these patients was collected by reviewing medical records and electronic records.

As this is a retrospective observational study, the results were collected in a completely anonymous and aggregated form; therefore, the acquisition of consent as indicated by the Privacy Guarantor is not expected.

## 5. Conclusions

Pertussis is a re-emerging disease even in upper-middle-income countries, showing that pertussis continues to be a significant public health challenge. Therefore, it is crucial to improve disease surveillance, management, and immunization so as to effectively address this challenge while avoiding the development of critical cases. Early diagnosis and treatment are important in clinical practice to promptly decide on the most appropriate case management by allowing specific antibiotic treatment to be started immediately. In unvaccinated or partially immunized individuals such as infants, pertussis is associated with significant morbidity and, in rare cases, mortality. This highlights the need to implement a vaccination campaign and to monitor the immunological status of pregnant women and caregivers so as to prevent the increase in pertussis cases.

## Figures and Tables

**Table 1:** Demographic, microbiological, and clinical characteristics.

	Patient 1	Patient 2	Patient 3	Patients 4	Patient 5
Sex	M	M	M	M	F
Age, years	16	14	0.11	0.08	0.07
Nationality	Italian	Italian	Italian	Italian	Italian
*B.pertussis* vaccination	NA	NA	No	No	No
Temperature (celsius)	36.5	36.4	36.5	36.6	37
Diagnosis:	Molecular	Molecular	Molecular and culture	Molecular and culture	Molecular and culture
*B. pertussis* DNA	Positive	Positive	Positive	Positive	Positive
cycle threshold	26	30	24	22	9
*B. pertussis* culture	Negative	Negative	Positive	Positive	Positive
Coinfection	-	Human Rhinovirus	*Haemophilus influenzae* *Staphilococcus aureus* *Enterobacter cloacae*	Rhinovirus/Enterovirus Respiratory Syncytial virus	Rhinovirus/Enterovirus *Staphilococcus aureus*
Clinical manifestation	Laryngospasm, cough, dyspnea	Cough	Cough bronchiolitis	Cough oxygen desaturation	Respiratory failure
Respiratory symptoms before admitted, day	9	10	5	10	Na
Hospitalization	Yes	No	Yes	Yes	Yes
Day of hospitalization	7	-	26	11	Na
Antimicrobial therapy	Azitromycin Clarithromycin	Clarithromycin	Clarithromycin	Clarithromycin	Meropenem Vancomycin Azitromycin
Other treatment	Cortisone	Na	Immunoglobulin corticosteroid	Cortisone low-flow oxygen	Mechanical ventilation Extra-corporeal membrane oxygenation (ECMO)
Outcome	Clinical cure	Na	Clinical cure	Clinical cure	Dead

Na: not available.

## Data Availability

The original contributions presented in the study are included in the article; further inquiries can be directed to the corresponding author.

## Supplementary Materials

The following supporting information can be downloaded at: https://www.mdpi.com/article/10.3390/antibiotics13050464/s1, Supplementary File S1: Timeline portraying the patient’s microbiological and clinical history.

## References

[1] Guiso N. (2014). Bordetella pertussis: Why is it still circulating?. J. Infect..

[2] Di Camillo C., Vittucci A.C., Antilici L., Ciarlitto C., Linardos G., Concato C., Lancella L., Villani A. (2021). Pertussis in early life: Underdiagnosed, severe, and risky disease. A seven-year experience in a pediatric tertiary-care hospital. Hum. Vaccin. Immunother..

[3] Long S., Lowe R.B. (2022). Severe Pertussis Infection With Hyperleukocytosis in a 10-Month-Old Unvaccinated Amish Female: A Case Report. Cureus.

[4] Carbonetti N.H. (2016). Pertussis leukocytosis: Mechanisms, clinical relevance and treatment. Pathog. Dis..

[5] Liao Y., Li W.R., Zhu Y., Luo S.H., Liao Q., Wan C.M. (2022). Invasive Bordetella pertussis Infection in Infants: A Case Report. Open Forum Infect Dis..

[6] Madi M.Y., Shahwan M.Y., Nayar C., Kher S. (2019). Coughing Up a Lung: A Curious Case of Pertussis. Cureus.

[7] Jiang W., Wu M., Chen S., Li A., Wang K., Wang Y., Chen Z., Hao C., Shao X., Xu J. (2021). Virus Coinfection is a Predictor of Radiologically Confirmed Pneumonia in Children with Bordetella pertussis Infection. Infect. Dis. Ther..

[8] Jiang W., Mao L., Wang K., Wang Y., Hao C., Shao X., Xu J. (2021). Prevalence of *B. pertussis* infection in children with clinically suspected pertussis. J. Microbiol. Immunol. Infect..

[9] Esposito S., Principi N. (2018). Prevention of pertussis: An unresolved problem. Hum. Vaccin. Immunother..

[10] Pertussis—Number of Reported Cases. https://www.who.int/data/gho/data/indicators/indicator-details/GHO/pertussis-number-of-reported-cases.

[11] Surveillance Atlas of Infectious Diseases. https://atlas.ecdc.europa.eu/public/index.aspx?Dataset=27&HealthTopic=38.

[12] Kaur G., Danovaro-Holliday M.C., Mwinnyaa G., Gacic-Dobo M., Francis L., Grevendonk J., Sodha S.V., Sugerman C., Wallace A. (2023). Routine Vaccination Coverage—Worldwide, 2022. MMWR Morb. Mortal. Wkly. Rep..

[13] https://www.epicentro.iss.it/vaccini/dati_Ita#pertosse.

[14] Shi J., Wang C., Cui Y., Zhang Y. (2018). Extracorporeal membrane oxygenation with prone position ventilation successfully rescues infantile pertussis: A case report and literature review. BMC Pediatr..

[15] Birru F., Al-Hinai Z., Awlad Thani S., Al-Mukhaini K., Al-Zakwani I., Al-Abdwani R. (2021). Critical pertussis: A multi-centric analysis of risk factors and outcomes in Oman. Int J Infect Dis..

[16] Domic M., Ridout D., MacLaren G., Barbaro R., Annich G., Schlapbach L.J., Brown K.L. (2018). Extracorporeal Membrane Oxygenation for Pertussis: Predictors of Outcome Including Pulmonary Hypertension and Leukodepletion. Pediatr. Crit. Care Med..

[17] Fueta P.O., Eyituoyo H.O., Igbinoba O., Roberts J. (2021). Cardiopulmonary Arrest and Pulmonary Hypertension in an Infant with Pertussis Case Report. Case Rep. Infect. Dis..

[18] Halasa N.B., Barr F.E., Johnson J.E., Edwards K.M. (2003). Fatal pulmonary hypertension associated with pertussis in infants: Does extracorporeal membrane oxygenation have a role?. Pediatrics.

[19] Klein N.P., Bartlett J., Rowhani-Rahbar A., Fireman B., Baxter R. (2012). Waning protection after fifth dose of acellular pertussis vaccine in children. N. Engl. J. Med..

[20] Ulloa-Gutierrez R., Boza R., Carvajal-Riggioni D., Baltodano A. (2011). Pertussis: Should we improve intensive care management or vaccination strategies?. Expert Rev. Vaccines.

[21] Fiasca F., Necozione S., Mattei A. (2021). Pertussis in Italy: How to protect the “unprotectable”?. Hum. Vaccin. Immunother..

