# Supplementary material for: Whooping Cough Cases Increase in Central Italy after COVID-19 Pandemic
Source: Antibiotics (Basel). 2024 May 19;13(5):464. doi: 10.3390/antibiotics13050464 (PMC11118037; doi:10.3390/antibiotics13050464)
Supplement: Supplementary file 1 [file antibiotics-13-00464-s001.zip › antibiotics-2975184-supplementary.pdf]

Patient #1

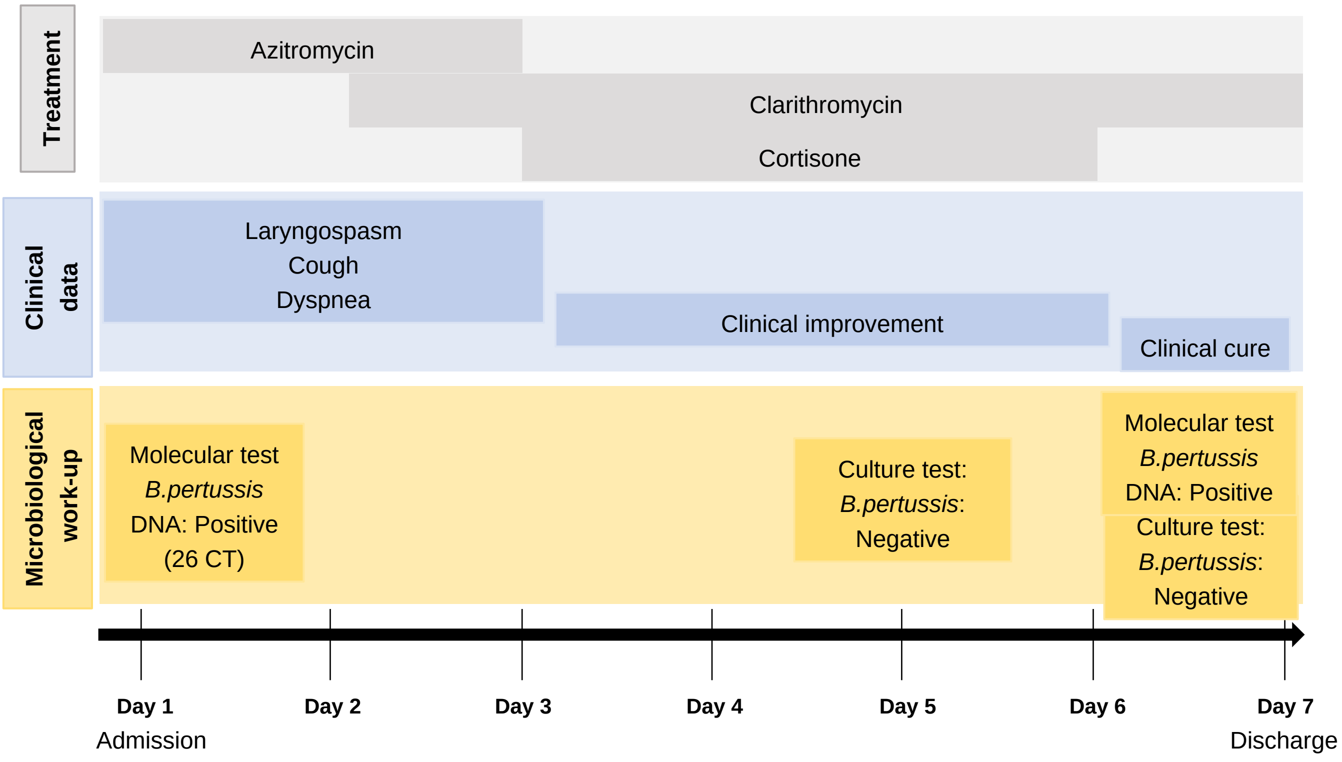

Patient #2

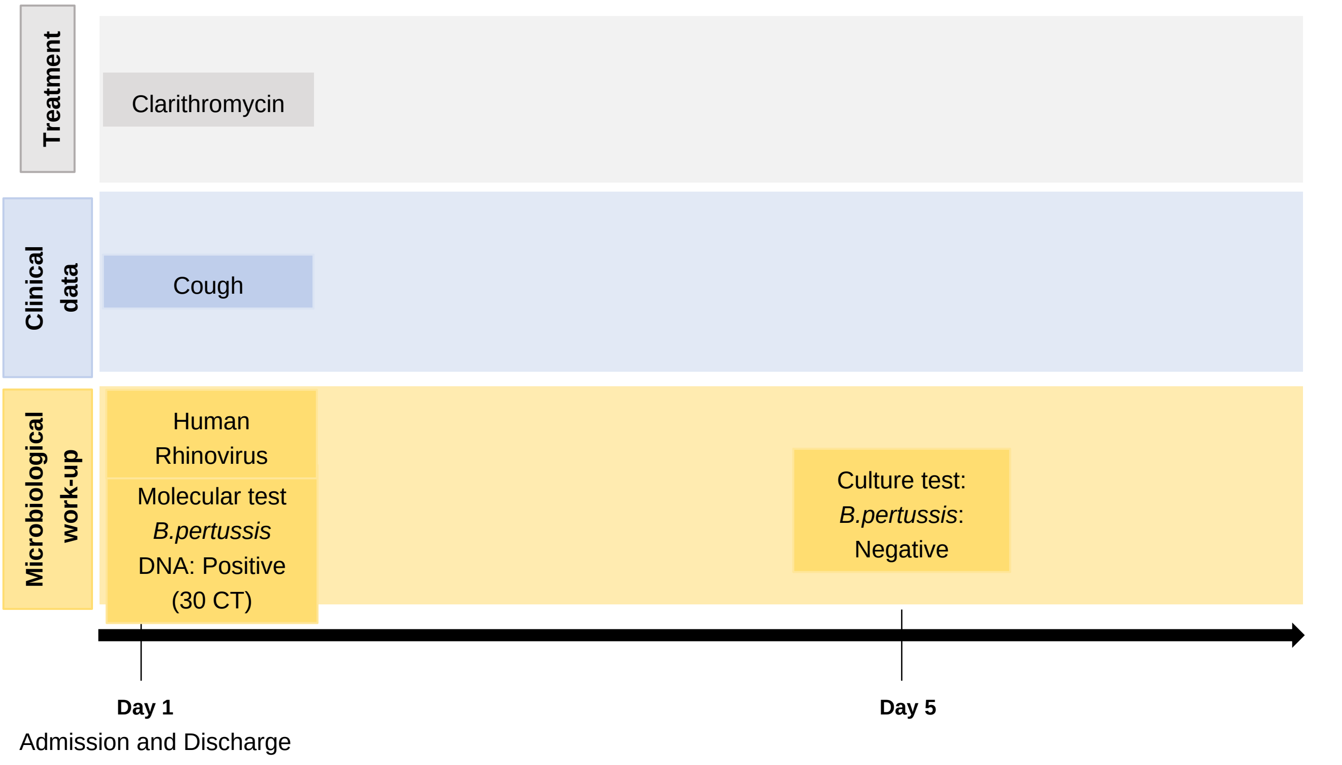

Patient #3

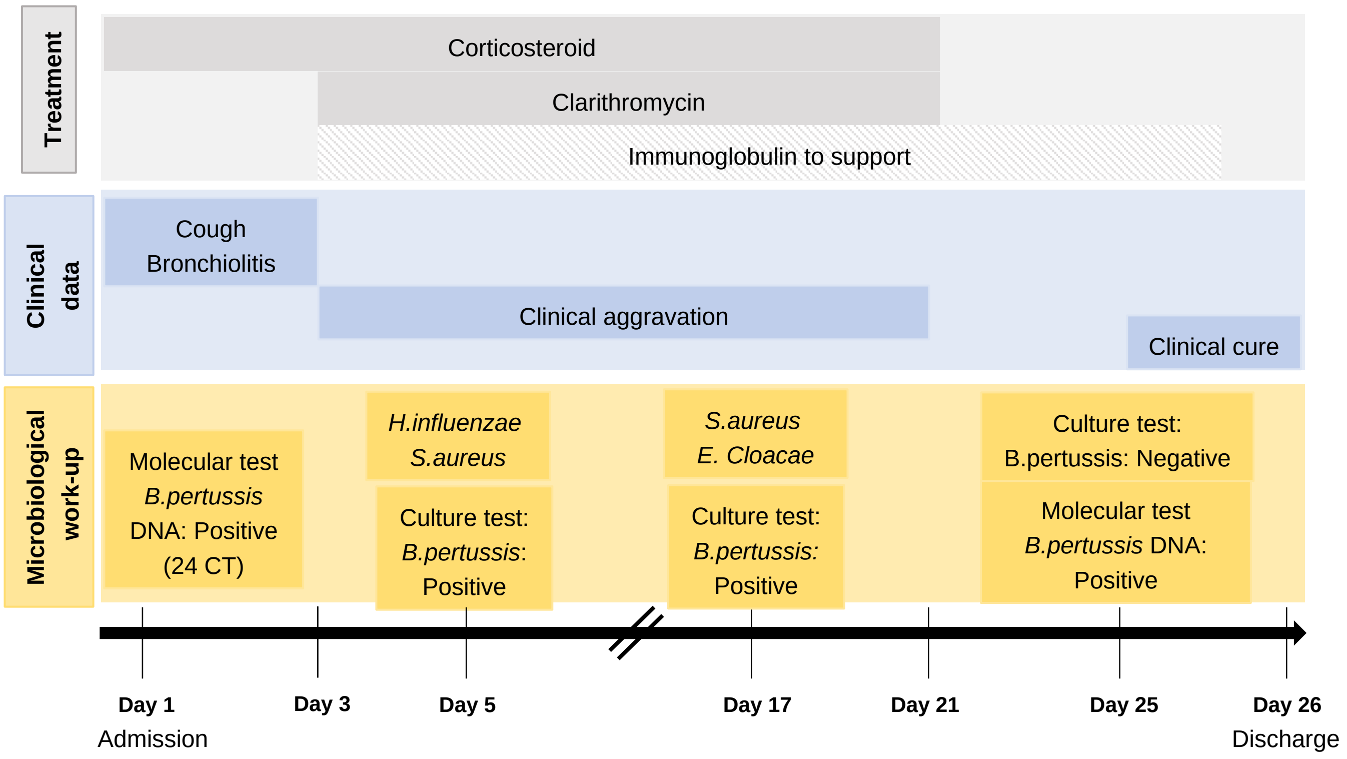

Patient #4

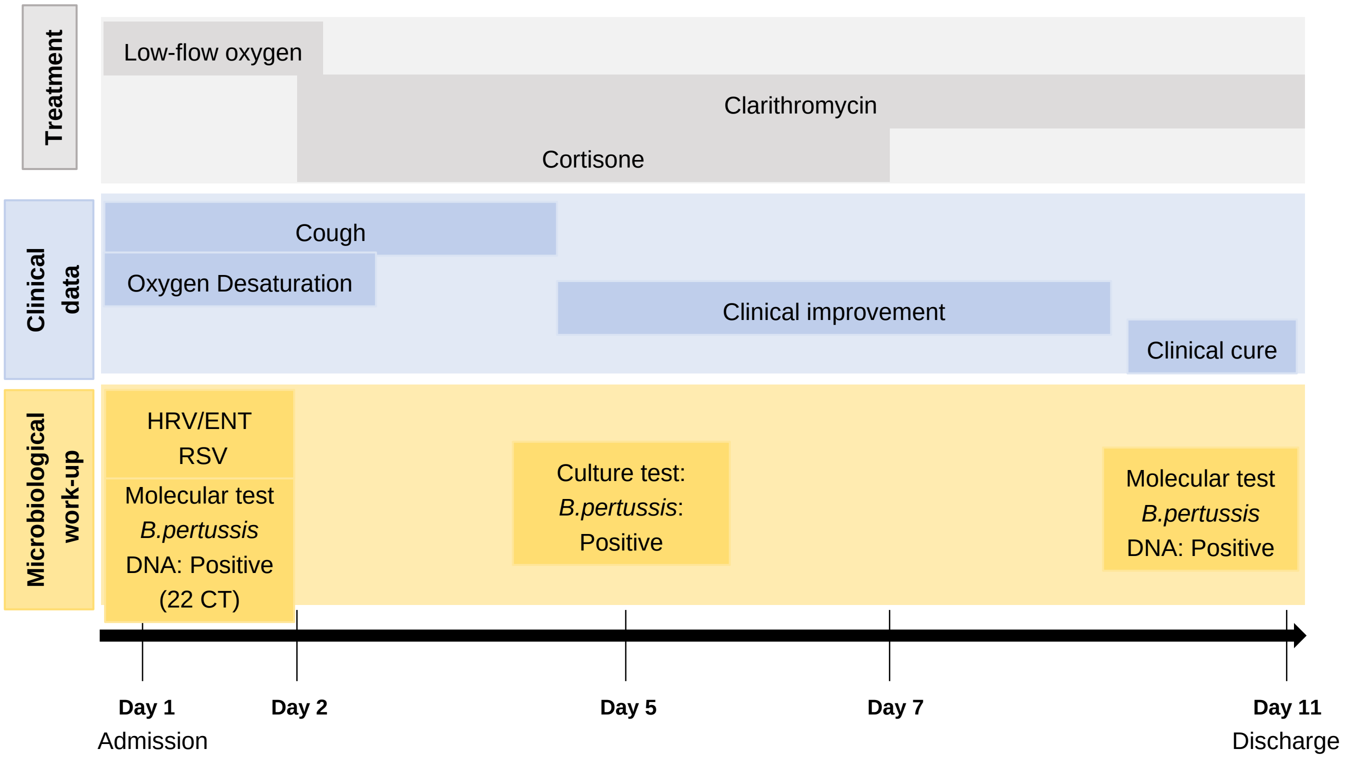

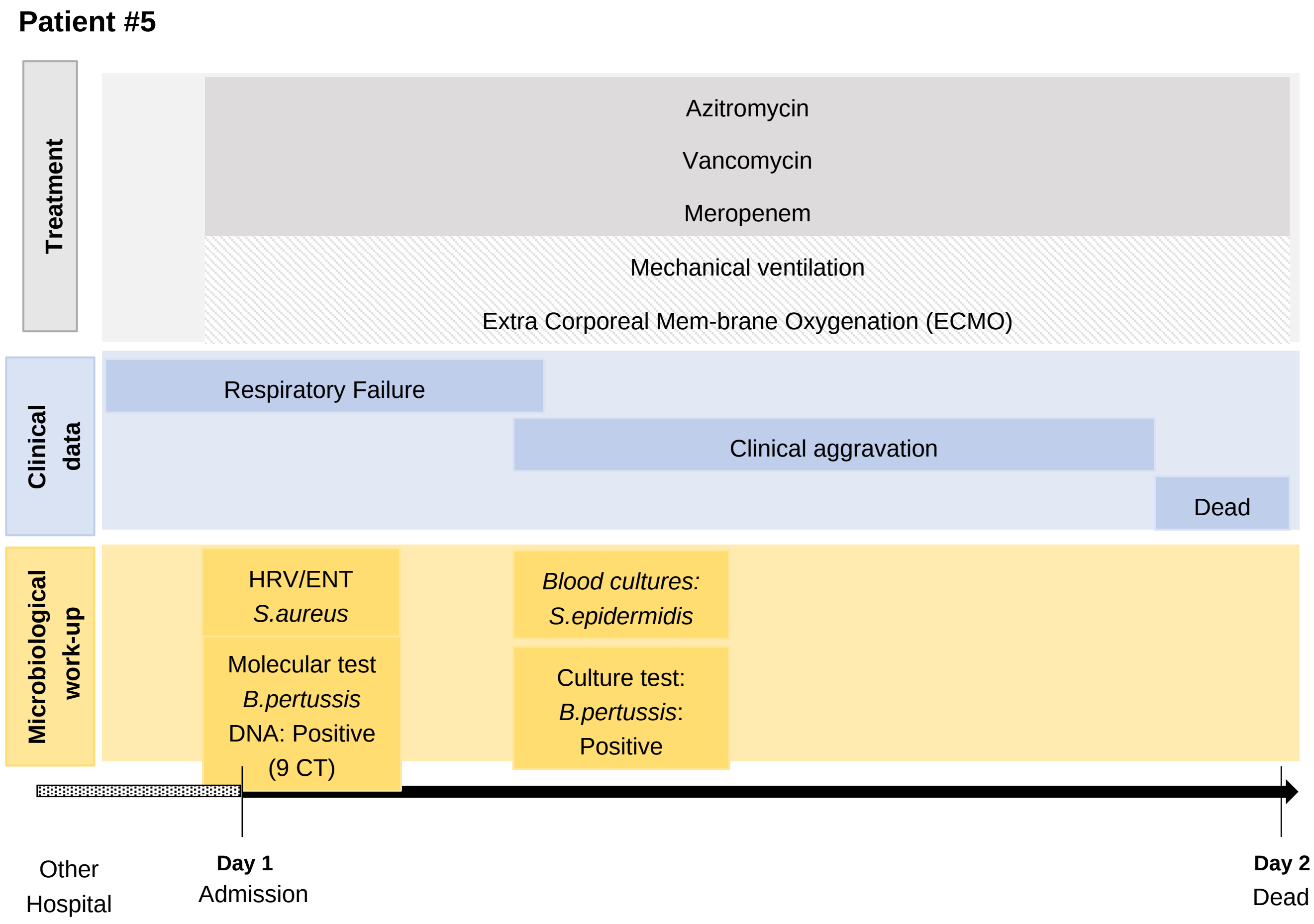

Supplementary File S1: Timeline portraying the patient's microbiological and clinical history. Abbreviation: CT: cycle threshold; HRV/ENT: Rhinovirus/Enterovirus; RSV: Respiratory Syncitial virus.
